# The Use of Microalgae for Coupling Wastewater Treatment With CO_2_ Biofixation

**DOI:** 10.3389/fbioe.2019.00042

**Published:** 2019-03-19

**Authors:** Marziyeh Molazadeh, Hossein Ahmadzadeh, Hamid R. Pourianfar, Stephen Lyon, Pabulo Henrique Rampelotto

**Affiliations:** ^1^Faculty of Engineering, Department of Civil Engineering, Ferdowsi University of Mashhad, Mashhad, Iran; ^2^Faculty of Science, Department of Chemistry, Ferdowsi University of Mashhad, Mashhad, Iran; ^3^Culture and Research (ACECR)-Khorasan Razavi Branch, Industrial Fungi Biotechnology Research Department, Academic Center for Education, Mashhad, Iran; ^4^SRL-Environmental, LLC, Racine, WI, United States; ^5^Center of Biotechnology and PPGBCM, Federal University of Rio Grande do Sul, Porto Alegre, Brazil

**Keywords:** microalgae, carbon dioxide biofixation, wastewater treatment, biomass production, global warming

## Abstract

Production and emission of CO_2_ from different sources have caused significant changes in the climate, which is the major concern related to global warming. Among other CO_2_ removal approaches, microalgae can efficiently remove CO_2_ through the rapid production of algal biomass. In addition, microalgae have the potential to be used in wastewater treatment. Although, wastewater treatment and CO_2_ removal by microalgae have been studied separately for a long time, there is no detailed information available on combining both processes. In this review article, microalgae-based CO_2_ biofixation, various microalgae cultivation systems,¯ and microalgae-derived wastewater treatment are separately discussed, followed by the concept of integration of CO_2_ biofixation process and wastewater treatment. In each section, details of energy efficiency and differences across microalgae species are also given.

## Introduction

Global warming is one of the major concerns for many countries around the world. This issue has mainly been attributed to high concentrations of gases where carbon dioxide (CO_2_) is the largest contributor, being responsible for up to 60 percent of the total greenhouse gases (E. I. A. U.S. Department of Energy, 1997; Yamasaki, 2003; Coyle, 2007). CO_2_ concentration in the atmosphere has increased from pre-industrial levels of 280 ppm to about 400 ppm today^1^ A major part of this noticeable increase lies in the growing demands for fossil fuels in energy and transportation sectors. It is anticipated that atmospheric CO_2_ levels will continue to rise to about 570 ppm by Twenty-Second century (Stewart and Hessami, 2005). Consequently, the world temperature could increase by 1.9°C, while the sea level could experience an average increase of 3.8 m (Stewart and Hessami, 2005). Due to increasing concerns about the greenhouse gases-caused climate change, the Kyoto protocol was established as the first agreement between world nations to mandate country-by-country reductions in greenhouse gas emissions at 1997.

Since CO_2_ is the largest contributor to the greenhouse effect, a reduction in CO_2_ level will directly affect the total greenhouse gas emissions. Currently, three major methods are being taken into action in order to remove excess atmospheric CO_2_: (i) the use of chemical reactions including chemical/physical solvent scrubbing, adsorption, cryogenics, and membranes, (ii) The storage of CO_2_ emitted underground or into the ocean (Kumar et al., 2010), and (iii) Transforming CO_2_ to organic matters through biological mitigation.

Chemical methods are not environmentally sustainable and require considerable space and investment. In addition, the most noticeable challenge with the storage method could be the potential for CO_2_ leakage over the years (Lackner, 2003). Accordingly, the biological mitigation is an economically practical and environmentally sustainable technology which has achieved much attention as an alternative method in the long term (Kumar et al., 2010). Biological CO_2_ fixation usually occurs through photosynthesis by terrestrial plants and trees. However, they are able to eliminate only 3–6 percent of CO_2_ because of their slow growth, while other microorganisms such as eukaryotic algae and cyanobacteria can fix CO_2_ 10–50 times faster (Iasimone et al., 2017). Algae are capable of eliminating 513 tons of CO_2_ and producing up to 100 tons of dry biomass per hectare per year (Bilanovic et al., 2009). Another advantage with algae lies in the production of renewable fuels such as biodiesel and hydrogen. Since CO_2_ emitted during combustion of these biofuels can be assimilated by the algae, there is a resulting net zero balance of CO_2_ emissions (Kumar et al., 2010). Furthermore, algae can be a source of nutrition (Sabra et al., 2001; Becker, 2007; Plaza et al., 2008; Raja et al., 2008; Dvir et al., 2009; Gressler et al., 2010) including vitamins (Lee, 2001; Raja et al., 2008), minerals (Lee, 2001; Dvir et al., 2009), and proteins (Raja et al., 2008; Dvir et al., 2009) which in turn may help to offset the cost of CO_2_ bioremediation.

Microalgal species can treat municipal, industrial, agro-industrial, and livestock wastewaters. Microalgal systems for the treatment of other wastes such as the effluent from food processing and other agricultural wastes have been also reported in the literature. Furthermore, algae-based strategies for the removal of toxic minerals such as As, Br, Cd, Hg, Pb, Sc, and Sn ions have also been reported individually or in a mixture (Abdel-Raouf et al., 2012).

Many microalgae species have been adapted to grow efficiently in wastewater. This way, the cost of production may be decreased due to the simultaneous use of wastewater and cultivation of specific nutrient-rich microalgae. Therefore, microalgae-mediated CO_2_ bio-mitigation can be more economic, cost-effective, and eco-friendly, when it is incorporated into a wastewater treatment infrastructure (Kuo et al., 2016; Collotta et al., 2018).

In this chapter, several issues related to the use of microalgae for CO_2_ biofixation and wastewater treatment are discussed. Details with regard to CO_2_ removal techniques, microalgae cultivation systems, wastewater treatment methods, and evaluation of the integration of carbon capture technology to wastewater treatment are provided.

## Carbon Dioxide Removal Strategies

### Non-biological Methods

#### Carbon Dioxide Sequestration

By definition, CO_2_ sequestration is a long-term CO_2_ process in order to decrease the level of CO_2_ emitted to the atmosphere. In this regard, there are various storage approaches that could be taken into account, including: mineralization, ocean storage, and geological storage. These storage methods also should comply with the following specifications: (i) safe storage, (ii) minimal environmental impact (iii) verifiable storage (iv) indefinite storage (Lackner and Brennan, 2009).

Mineralization is a process by which gaseous CO_2_ is transformed to solid inorganic carbonates by chemical reactions. Among others, calcium and magnesium silicates can uptake CO_2_ from the atmosphere through the process of carbonate formation, leading to a permanent storage of the CO_2_. This process offers a sustainable opportunity to safely store CO_2_ during a long period. However, it is obviously very slow in nature and thus a high cost of technology may be needed to speed up this process (Allen and Brent, 2010).

The ocean is considered to be the largest natural reservoir of CO_2_ (Khoo and Tan, 2006) so that the injection of all the anthropogenic CO_2_ would change the ocean carbon concentration and pH by less than 2% and 0.15 units, respectively (Herzog and Golomb, 2004). The natural ocean storage process consists of transporting captured CO_2_ to the deep ocean. However, concerns have been raised by environmentalists with regard to environmental impacts of CO_2_ stored in the ocean, including serious effects on marine life and acidification of water. The mass discharges of CO_2_ are considered by some to be the equivalent of uncontrolled dumping of toxic waste into the world oceans. (Herzog and Golomb, 2004)

The option of storing CO_2_ beneath the earth's surface holds greater potential for controlled conditions with the least environmental impact. The storage locations could include saline aquifers, spent oil and natural gas wells that are no longer in production and unmineable coal seams (Celia et al., 2009). Given the fact that human activity generates almost seven gigatons of carbon (GtC) per year, this storage option could accommodate hundreds to thousands of total GtC stored beneath the earth. (1 GtC = 1 billion metric tons of carbon equivalent) (Herzog and Golomb, 2004).

There are direct environmental and human health risks raised by environmentalists, including leaks, slow migration and accumulation and induced seismicity.

#### Carbon Dioxide Capture Approaches

For the past 40 years, fossil fuels have been blamed as the source for generating a significant portion of the anthropogenic CO_2_ in the atmosphere. Therefore, it is reasonable to capture CO_2_ from fossil fuels-dependent industrial operations in order to significantly decrease the atmospheric CO_2_ levels. The captured CO_2_ could be used in different industries, such as urea production, foam blowing, carbonating beverages, and the production of dry ice. Currently, the three main approaches of CO_2_ capture are (Pires et al., 2011): post-combustion, oxy-fuel combustion, and pre-combustion.

- Pre-combustion
  Pre-combustion is normally applied in integrated gasification combined cycle (IGCC) power plants. The process uses a high-pressure gasifier to convert coal into pressurized gas, composed of CO and H_2_. The CO_2_ will be captured from the synthesized gases, releasing H_2_ to be used further as the fuel in a gas turbine to produce electricity (Pachauri and Reisinger, 2007). A major limitation with this method is that the cost of electricity generated in pulverized coal (PC) power plants is lower than the costs in IGCC plants. An additional difficulty is that the pre-combustion process occurs only where methane is the main fuel.
- Oxy-Fuel Combustion
  In oxy-fuel combustion, the fuel is mixed with pure oxygen in a nitrogen free environment that results in a flue exhaust gas containing CO_2_ and water vapor. This way, the captured CO_2_ can be stored with less downstream processing.
- Post-combustion
  Among the CO_2_ capture technologies, the time needed for the development of coal-derived
  Syngas separation, hydrogen turbine, and fuel-cell technologies significantly decreases if a post-combustion method is implemented. Unlike pre-combustion, post-combustion can be fitted to fossil fuel-fired power plants (Pires et al., 2011). Presently, post-combustion capture can be performed employing several technologies as explained below (Pires et al., 2011).

◦Adsorption
  Adsorption includes diffusing molecules, atoms, or ions of a gas or a liquid to the surface of a solid, where they may bond with the solid surface or establish weak intermolecular forces. The Intermolecular forces between gases and the surfaces of certain solid materials would be the main process in CO_2_ adsorption. This is dependent on various factors such as temperature, partial pressures, surface forces, adsorbent pore sizes, and the selectivity of the adsorbent material (Xiao et al., 2008).
  Adsorption can be processed through either chemical bonds or physical forces. Chemical bonds are composed an immobilized amine or other reactants on a support surface. The adsorbent reacts with the CO_2_ in the flue gas (Pires et al., 2011). Physical adsorption works based on the intensity of intermolecular forces, where the forces between molecules of a solid and the flue gas are greater than those between molecules of the gas itself (Pires et al., 2011).
  Thus far, several chemical and physical materials have been proposed to serve as adsorbents, including carbon fiber monolithic adsorbents (Thiruvenkatachari et al., 1997), activated carbon fiber-phenolic resin composites, melamine–formaldehyde highly porous adsorbent, and amine immobilized adsorbents (Meisen and Shuai, 1997).
◦Membrane separation
  With regard to CO_2_ removal, two major kinds of membranes are normally used; gas separation and gas absorption membranes. Gas separation contains solid membranes that operate based on of a porous structure that allows the preferential permeation of mixture constituents (Meisen and Shuai, 1997). The separation process is a function of both time and the physical/chemical properties of the membrane that favors one part of the gas mixture over the other at a fixed rate of gas permeation.
  CO_2_ is taken up on one side of the membrane, diffuses through it and is released on the other side (Pires et al., 2011). Gas absorption membranes are micro porous solid membranes that need to be in contact with an adsorbent, such as a liquid (Meisen and Shuai, 1997). The absorption liquid on one side of the membrane selectively takes up CO_2_ from the mixed gas stream on the other side of the membrane (Pires et al., 2011).
  Gas absorption membranes can be grouped into polymer membranes, palladium membranes, and molecular sieves (Pires et al., 2011).
◦Cryogenic separation
  A temperature-based approach can be used for the select removal and concentration of CO_2_ (Pires et al., 2011). The use of cryogenic processes, also known as low temperature distillation, to remediate flue gas emission is still at its early stages. This involves cooling and liquefying concentrated CO_2_ in a stream gas through several stages to eventually induce phase changes in CO_2_ (Meisen and Shuai, 1997). This method is more effective for gases stored at ambient or lower temperature with high concentrations (>50%). Thus, it may not be economically recommended for diluted gases containing CO_2_ such those emitted by coal-fired plants (Herzog and Golomb, 2004).
◦Absorption
  Carbon dioxide can be removed through an absorption system, comprised of two components; the absorber and the stripper (desorber) (Erga et al., 1995). The absorption system operates on physical, chemical or hybrid absorption processes. In chemical absorption, CO_2_ is absorbed by a liquid solvent that forms a temporary chemical bond with the gas. CO_2_ then reacts with one or more basic chemical absorbent to form a weakly bonded intermediate compound. This compound is then broken down by heat, regenerating the original absorbent and producing a CO_2_ stream (Erga et al., 1995). In industrial settings, CO_2_ separates from the gas stream by bubbling into a liquid absorbent in a packed absorber column. The absorbed CO_2_ is stripped out of the chemical solvent by a counter flowing steam at 100–120°C passing through a regenerator unit. In order to remove water vapor from the stream it should be condensed resulting in the highly concentrated (over 99%) CO_2_. The solvent is cooled to 40–65°C and recycled into the absorption column. Typical absorbents commercially available in a chemical absorption system include amine carbonate-based components such as mono-ethanolamine (MEA), diethanolamine (DEA), ammonia and hot potassium carbonate (Erga et al., 1995).
  According to Henry's law, physical absorption-based CO_2_ removal is performed through physically absorption to a solvent followed by regeneration applying heat and/or pressure reduction. Some of the commercially available physical absorbents include Selexol (dimethylether of polyethylene glycol) and Rectisol (cold methanol) which are applied at high pressure.
  In addition to the physical or chemical absorption, a combination of the best characteristics of both methods can also be utilized, known as hybrid absorption. Several commercial absorbent have been used to serve as hybrid absorption, including Sulfinol (a composite solvent, including a mixture of diisopropanolamine (30–45%) or methyl diethanolamine (MDEA), sulfolane (tetrahydrothiophene dioxide) (40–60%), and water (5–15%) (Desideri and Corbelli, 1998).

### Carbon Dioxide Biofixation Using Microalgae

Near half of the dry biomass weight of microalgae is made of carbon, thus the carbon content in a microalgae growth medium in dissolved or gaseous form is crucial and often a limiting factor for maximal productivity. The concentration of CO_2_ in the atmosphere (only about 0.04% *v/v*) is not sufficient to provide carbon for algal growth. Microalgae are naturally able to attain their carbon from several other sources, including CO_2_ from industrial flue gases, and those chemically fixed in soluble carbonate compounds (e.g., NaHCO_3_ and Na_2_CO_3_)(He et al., 2016). The main concept is that microalgae utilize CO_2_ as its main carbon source for a wide array of metabolic processes. Waste gases from combustion represent a viable source of CO_2_ that can be directly introduced into large-scale microalgae production systems, as they usually contains CO_2_ in volume fraction of 5 to 15%. In addition, the CO_2_ converted in the form of algal biomass can be further used as food, feed, fertilizer, or fuel. Processes including digestion, respiration and combustion of the biomass could release CO_2_ back into the air. Thus, it would be logical to assume that microalgae are not simply CO_2_ reservoir but they act like a biological post-combustion tool for capturing CO_2_ from flues gases emitted from power plants.

Microalgae-derived CO_2_ biofixation can promote production of valuable algal biomass and simultaneously reduce greenhouse gas emissions. However, little progress has been made to lower the cost of this approach (Herzog and Golomb, 2004). The most important advantage of CO_2_ fixation by microalgae is their rapid and widespread growth. In addition, biofuel produced by microalgae is highly biodegradable and contains no sulfur or toxic materials (Demirbas, 2011).

Tolerance to the CO_2_ concentrations of a flue gas has been shown to be different across different microalgae species. Limited groups of microalgae have shown extraordinary tolerance of high concentration of CO_2_, including *Chlorella* spp., *Arthrospira* (formerly *Spirulina*) spp., *Scenedesmus dimorphus, Botryococcus braunii*, and *Nannochloropsis oculate* (Cheah et al., 2015). Under high CO_2_ concentrations (10–80%), *Scenedesmus* spp. showed more tolerance than *Chlorella* spp., while both species were able to grow in lower CO_2_ concentrations (10–30%). However, *Scenedesmus* spp. growth was prevented under 100% CO_2_ concentration (Hanagata et al., 1992). A mutant of *Chlorella* spp. (strain KR-1) was reported to grow even under CO_2_ levels as high as 70% (Sung et al., 1998). Flue gases from a coke oven, hot stove, and power plant in a steel plant was used for *Chlorella* sp. MFT-15 cultivation. The maximum average specific growth and lipid production were reported to be 0.77/day and 0.81 g/L, respectively. The findings revealed that *Chlorella* sp. MFT-15 could efficiently utilize the CO_2_, NO_X_, and SO_2_ present in the different flue streams (Kao et al., 2014). Another study showed an excellent tolerance in *S. dimorphus* to stream gas containing high levels of CO_2_ (2–20%), NO (150–500 ppm), and SO_2_ (100 ppm)(Jiang et al., 2013).

The rate of CO_2_ fixation by microalgae might also be different across various species or even mutated strains of the same species. Evaluation of five different microalgae suitable for mass cultivation showed significant differences in CO_2_ fixation rates as follows: *Dunaliella tertiolecta* SAD-13.86 (272.4 mg/L/day), *Chlorella vulgaris* LEB-104 (251.64 mg /L/day), *Spirulina platensis* LEB-52 (318.61 mg/L/day), *Botryococcus braunii* SAG-30.81 (496.98 mg /L/day), and *Chlorococcum littorale* (1,000 mg/L/day) (Sydney et al., 2010). While *C. vulgaris* fixed CO_2_ emitted from flue gas up to 260 mg/L/h (Larsson and Lindblom, 2011), *C. sorokiniana* yielded 330 mg L^−1^ after 96 h of exposure to flue gas containing CO_2_(Lizzul et al., 2014). It was also found that a mutant strain of *Scenedesmus obliquus* WUST4 captured CO_2_ from flue gas and accumulated a high biomass concentration (0.922 g/ L) under 10% CO_2_ concentrations, as compared to that of the non-mutant *S. obliquus* (0.653 g/ L) under higher concentration of CO_2_ (20%) (Li et al., 2011).

## Microalgae Cultivation System Technology

The type of final products, the source of nutrients, the criteria for cultivation (CO_2_ capture, biofuels, etc.) and the capital cost (CC) and operation and maintenance (O&M) are the driving forces behind the diversity in cultivation systems described in the literature. The cultivation systems of microalgae could be classified into two major conditions: “open” or “closed” systems, even though sometimes these groups might overlap. In essence, the “open” systems (such as ponds, lagoons, and deep channels) are usually established outdoors. The “closed” systems are usually located indoor under artificial light or outdoors under sunlight and should include vessels or tubes with walls made of transparent materials. These cultivation systems and the industrial scale production for each system have been compared and summarized in Table 1.

**Table 1:** Microalgae commercial culture systems and their applications to wastewater treatment.

**Production system**	**Prospects**	**Limitation**	**Company**	**Scale^a^**	**Application to wastewater treatment**	**References**
Open systems	Easier to construct, operate, and clean up. Relatively economical after cultivation, Good for mass cultivation.	Poor light utilization, evaporative losses, diffusion of CO_2_ to the atmosphere, requirement of large areas of land, limited to few strains of algae, cultures Easily contaminated	LiveFuels	Demonstration (18.2 ha site)	Possible applications to wastewater treatment mentioned	a
			Kent BioEnergy	Full (64.7 ha site)	Demonstrated or specifically intended for WWT	(Brune et al., 2007; Ugwu et al., 2008; Schwartz et al., 2010; Wu et al., 2010a,b)
			Algaeventure Systems	Pilot	Possible applications to wastewater treatment mentioned	a
Tubular photobioreactors	Suitable for outdoor, good biomass productivities, relatively inexpensive	High levels of dissolved oxygen (DO), adverse pH and CO_2_ gradients, fouling, photoinhibition is very common in outdoor tubular photobioreactors	Solix Biofuels	Pilot (0.8 ha site)	Possible applications to wastewater treatment mentioned	a
			A2BE carbon capture	Bench	Possible applications to wastewater treatment mentioned	(Torzillo et al., 1986; Richmond et al., 1993; Molina et al., 2001; Vonshak and Torzillo, 2004; Sears, 2007; Willson et al., 2008, 2009; Wilkerson and Watters, 2009; Wilkerson et al., 2009)
			Bionavitas	Bench	Possible applications to wastewater treatment mentioned	a
Flat panel photobioreactor	Modular design makes it easy to scale up production, low accumulation of dissolved oxygen and high concentration of sunlight per square cm.	Temperature control issues, algal biofilm formation, strain-specific hydrodynamic stress issues	Bionavitas	Bench	Possible applications to wastewater treatment mentioned	(Richmond and Cheng-Wu, 2001; Ugwu et al., 2008; Wilkerson and Watters, 2009; Wilkerson et al., 2009; Xu et al., 2009)
Tic bag Photobioreactor	Low cost and good sterility	Disposal of used plastic bags may present a significant challenge at large scale operations,	Sapphire Energy	Pilot	Not mentioned	(Mendez et al., 2009a,b); (Fang et al., 2010; Hanin et al., 2010; Mendez et al., 2010; Wang et al., 2012)

a *According to information available on company website*.

### Open Systems

Historically, open ponds are used in commercial-scale algae production due to their low construction costs and ease of operation (Cai et al., 2013). In general, these systems can be classified into natural or artificial water systems. In natural water systems, algae production occurs in lakes, lagoons, and ponds. Artificial water systems occur in a variety of sizes, offer a greater degree of environmental controls and include man-made ponds, tanks, and various above ground and below ground containers (Ugwu et al., 2008).

In addition, different shapes, sizes, and types of open systems have been devised depending on the application (Tredici, 2004). Most importantly, open systems could be classified as non-stirred and stirred ponds in terms of aeration and distribution of nutrient in the media. In short, non-stirred ponds are more economical and simpler to manage, while stirred ponds provide proper aeration, light, and distribution of nutrients that in turn improve the growth of microalgae.

#### Non-stirred Ponds

Non-stirred ponds with an average depth of one half meter or less are the simplest of intermediate to large-scale culturing facilities. More than 30 tons per year (dry weight) of algae are harvested from non-stirred ponds and lakes in South-East Asia (Lee, 1997). Non-stirred ponds are subject to the same processes in natural ponds that include predation by zooplankton, mixed algal populations and well as the potential growth of pathogens (protozoans and bacteria) (Chaumont, 1993).

Accordingly, there are limited number of microalgae species, namely *Dunaliella salina* (Benemann et al., 1987; Borowitzka and Borowitzka, 1990) that have been grown commercially using this method.

#### Stirred Ponds

The most common forms of stirred ponds are high rate algal ponds (HRAP) and circular ponds. HRAP, also known as raceway ponds (Tredici, 2004) are open and shallow (15–25 cm deep) where the water is circulated with a paddlewheel. The raceways are constructed with either individual or multiple channels in a closed loop (Razzak et al., 2013). Some researchers obtain biomass concentrations of up to 1 g dry weight/L and 60–100 mg dry weight/L/d productivities in raceway ponds (Becker, 1994; Lee, 1997; Tredici, 2004). Raceway ponds are regularly utilized for the industrial culturing of *Chlorella* spp., *Arthrospira platen*sis, *Hematococcus* spp., and *D. salina* (Moheimani and Borowitzka, 2006). Generally, constructing raceways are still inexpensive compared to many forms of closed systems, nevertheless raceways suffer from low productivity because of algal contamination, poor mixing efficiency, inefficient use of CO_2_ and shading effect (Chisti, 2007; Mata et al., 2010).

In South-East Asia, circular ponds with a central pivot are used for the production of Chlorella spp. (Lee, 2001). These concrete ponds are commonly built with a depth of 25–30 cm and up to 45 m diameter while agitation is provided through a rotating arm. The algae are kept in suspension in 20–30 cm of nutrient-rich water with a paddlewheel. CO_2_ is bubbled into water to increase the total biomass yield due to the low solubility of atmospheric CO_2_ by the water.

### Closed Systems

Many of the problems associated with open-pond systems can be reduced or eliminated in closed pond systems, also known as bioreactors. The minimization of water evaporation and reduction of contaminating species are the main advantages of closed systems. While the problem of contaminating species is substantially reduced, photobioreactors cannot totally prevent the presence of other algal strains within the dominant population (Tredici, 2004). Though photobioreactor algal biomass production could be significantly improved, the major limiting factors for commercialization of closed systems are high capital and O&M costs.

Thus far, various types of photobioreactors have been designed for algae production.

#### Tubular Photobioreactor

Tubular photobioreactors, are the only type of closed systems which are employed for industrial purposes (Chisti, 2007). Common designs of vertical, horizontal, and helical tubular reactors are considered the easiest to scale up (Carvalho et al., 2006). In a common tubular photobioreactor, the algae and growth media are continuously circulated through the tubes to a reservoir using an airlift or mechanical pump (Razzak et al., 2013).

Pilot-scale tubular photobioreactors have been largely employed for growing a wide range of algae including *Arthrospira, Porphyridium, Chlorella, Dunaliella, Haematococcus, Tetraselmis*, and *Phaeodactylum* (Abdel-Raouf et al., 2012). Tubular photobioreactors have recurring problems including high levels of dissolved oxygen (DO) (Torzillo et al., 1986; Richmond et al., 1993; Molina et al., 2001), adverse pH and CO_2_ gradients. In addition, photoinhibition, especially when dealing with large scale applications, is a natural phenomenon occurring in outdoor photobioreactors (Vonshak and Torzillo, 2004).

#### Flat Plate Photobioreactor

The high degree of solar light on the surface of the plate, the lowered accumulation of dissolved oxygen and the convenience of modular design for scale-up are qualities that make flat-plate photobioreactors ideal for large-scale production in indoor and outdoor facilities. The drawbacks of flat plate photobioreactors include the ability to control the temperature, the formation of algal biofilms on the plates and the potential for hydrodynamic stress that could have a severe impact on certain strains of microalgae (Richmond and Cheng-Wu, 2001; Xu et al., 2009).

#### Plastic Bag Photobioreactor

Large bags nearly 0.5 meters in diameter and fitted with aerators are used as photobioreactors where they are hung vertically or placed in a metal or plastic cage for support and exposed to direct sunlight (Borowitzka, 1999; Tredici, 2004). In this system, the algae cultures are continuously mixed with diffused air pumped to the bottom of the bags (Tredici, 2004). The maintenance of this system demands frequent attention and the algae culture often crashes due to poor mixing.

## Harvesting and Biomass Extraction

Usually about 20 to 30% of the total production expenses for microalgae production is related to harvesting the biomass (Harun et al., 2011). The main problems in harvesting are the low biomass concentration in the open-pond cultivation system and the small cell size that makes the separation process consume a great deal of energy. Common methods of harvesting microalgae biomass include: coagulation, flocculation (or sedimentation), flotation (Harun et al., 2011),centrifugation (Molina Grima et al., 2003), membrane filtration (Lourenco et al., 2002; Feofilova et al., 2010), and ultrasonic separation (Bosma et al., 2003).

Through the coagulation and flocculation processes, polymers or salts are added to the culture to efficiently concentrate dispersed microalgae cells into larger aggregates such that they can be more easily removed from the open ponds by skimming or flotation. The disadvantage of this method is extra expense due to additional purification needed because coagulants are usually not desired in the subsequent lipid extraction step for biofuel production. Centrifugation is regarded as the most efficient method to harvest the biomass using mechanical forces, but their application in large-scale is limited because of the energy costs. Centrifugation can be cost-effective but it requires a two-step process. The typical concentration of algae in open pond cultivation is approximately 300 mg/L. Continuous-flow centrifuges work best if the source water has at least 1–3% solids. Gravity settling or dissolved air floatation are two simple methods to achieve the desired solids concentration. Membrane filtration has been applied in wastewater treatment involving removal of microalgae cell, which suffers some problems such as bio-fouling and also it is not cost-effective for large-scale biofuel production. Recently, ultrasonic separation (Varfolomeev et al., 2010) and electro-coagulation-flocculation (Vandamme et al., 2011) have been exploited for microalgae harvesting. Promising laboratory-scale results show that these methods are effective and have low-power consumption comparing to centrifugation, but full-scale testing is still in needed to evaluate their cost and performance. After harvesting, the next major challenge is the de-watering the concentrated biomass. Usually, 90% of the post-harvest weight of the microalgae paste is water which needs to be reduced below 50% by weight for efficient oil extraction (Reijnders, 2008). Water is removed by heating the biomass that could consume a great amount of energy. However, this process often uses waste heat from power plants to reduce the cost. It is crucial to minimize water contents after harvesting process to decrease energy demand and increase downstream extraction and fuel conversion efficiency.

## The Potential of Microalgae in Wastewater Treatment

As a consequence of the world's growing population, the increase of urban wastewater production has become one of many environmental challenges. Urban effluents have to be well-treated to an environmentally safe level before discharge into rivers, lakes, or the oceans (Arbib et al., 2012). Wastewater treatment may be processed at primary, secondary or tertiary levels using physical, biological, or chemical processes. Primary treatment removes settleable solids that can cause operational problems in advanced treatment steps. Secondary treatment is a physical/biological process that consumes the dissolved organic matter and oxidizes the major nutrients to nitrate and orthophosphate. Tertiary is an advanced treatment process that removes nitrates, phosphates and trace organic compounds (Droste, 1997).

In the urban wastewater treatment, the removal of macro-nutrients such as nitrates and phosphates is one of the main criteria for tertiary treatment. Usually, nitrogen is removed without further recycling and thus it is converted to N_2_ and will pass into the atmosphere (George et al., 2003; Muylaert et al., 2015). Phosphorus is mainly precipitated by the addition of cations such as calcium, aluminum, and iron which is costly (George et al., 2003). As an alternative to conventional tertiary treatment, both nitrogen and phosphorus can be removed by rapidly growing cultures of algae. This way, nitrogen and phosphorus can directly be taken up by microalgae, resulting in valuable algal biomass. The biomass can further be utilized as biofuel, feedstock or agricultural fertilizer. Composting the algal biomass with green waste (leaves, grass, husks, etc.) for 6 months will be sufficient to remove the pathogens found in wastewater.

### Use of Microalgae Strains With Special Attributes

Many species of microalgae are able to effectively remove nitrogen, phosphorus, heavy metals, pesticides, organic and inorganic toxins, and pathogens from wastewater. The main mechanism to remove pollutants includes accumulating and/or using them in their cells (Hoffmann, 1998). A number of studies have reported successful cultivation of several species of microalgae such as *Chlorella, Scenedesmus, Phormidium, Botryococcus, Chlamydomonas*, and *Arthrospira* for wastewater treatment and the efficacy of this method is promising (Olguì, 2003; Chinnasamy et al., 2010; Kong et al., 2010; Stephens et al., 2010; Pittman et al., 2011).

It should be noted that the efficiency of heavy metals removal depends on algal species. For example, chromium by *Oscillatoria* spp., cadmium, copper and zinc by *Chlorella vulgaris*, lead by *Chlamydomonas* spp., and molybdenum by *Scenedesmus chlorelloides* (Filip et al., 1979; Hassett et al., 1981; Sakaguchi et al., 1981; Ting et al., 1991). Furthermore, tolerance to organic pollutants in wastewater varies from species to species. *Euglena, Oscillatoria, Chlamydomonas, Scenedesmus, Chlorella, Nitzschia, Navicula*, and *Stigeoclonium* have been described as the most resistant genera to organic pollutants (Palmer, 1969).

### Systems of Algae-Based Waste Water Treatment

The two forms of algae culturing systems most commonly used for wastewater treatment are HRAP and waste stabilization ponds (WSPs). These are large shallow-water basins often used in temperate and tropical climates. A WSP is similar to a conventional oxidation pond where the raw wastewater is treated by a combination of algal and bacterial processes. The bacteria break down the complex organic matter into simpler compounds and the algae provide the oxygen necessary to run the aerobic bacterial processes. In conventional oxidation ponds, mechanical aerators provide the oxygen. The substitution of photosynthesis in place of electromechanical processes make WSPs a cost-effective, easily operated system for treating domestic and industrial effluent. This is especially significant with regards to wastewater treatment in tropical countries that often do not have a continuous supply of electricity.

Biochemical Oxygen Demand (BOD) is removed in the aerobic and anaerobic ponds in a WSP system. In the aerobic ponds, the algae produce oxygen that is used by the aerobic bacteria for the breakdown of complex organic matter. The residual BOD is removed in the anaerobic pond by heterotrophic bacteria including denitrifying bacteria. A final maturation pond plays a crucial role in the removal of human pathogens.

A low-cost approach to wastewater treatment and algae biomass production uses the HRAP system. This contains both a photobioreactor and intensified oxidation ponds where microalgae provide oxygen for bacteria, and in turn, bacteria convert mineral compounds (e.g., ammonium to nitrate) that supply nutrients for microalgae. This system is effective in reducing bacteria and BOD. Species of the genera *Oscillatoria, Micractinium, Arthrospira*, and *Scenedesmus* have been shown to grow well in HRAP systems and are relatively easy to harvest compared to smaller single-cell algae such as *Chlorella* spp. and *Oocystis* spp.

In traditional domestic wastewater, the ratios of carbon to nitrogen to phosphorus (C:N:P) are 20:8:1 while the C:N:P ratios for algae are 50:8:1. The additional carbon needed for algal photosynthesis is supplied from free carbon dioxide, which is the major limiting factor in HRAPs. Although high concentration of CO_2_ is generated by the bacteria in the ponds, a high photosynthetic rate can create a CO_2_ deficit. It is estimated that 30% of the total carbon required by microalgae can be supplied by sparging CO_2_ in these ponds (Craggs et al., 2013).

## The Integration of Wastewater Treatment to Carbon Capture Technology

### Concept of Integration

A proper medium for microalgae is supposed to provide sufficient nutrients that are needed for microalga growth. Wastewater combined with CO_2_ sparging creates an ideal growth medium for a wide variety of algae. However, in a domestic wastewater-based culturing system, the C:N ratio is not usually high enough to meet the requirements of algal biomass. In addition, atmospheric CO_2_ cannot sufficiently provide the needed carbon_._ Thus, the additional carbon could be supplied from other sources, such as flue gas (Larsson and Lindblom, 2011), which contains a high percentage of CO_2_ (Aaron and Tsouris, 2005). This CO_2_ addition can directly enhance the algal production that in turn can improve the efficiency of wastewater treatment. Previous publications have stated that the addition of CO_2_ to HRAP fed with domestic wastewater can remove nutrients to a level of treatment similar to those achieved by mechanical treatment technologies (Woertz et al., 2009). Furthermore, combining the wastewater nutrient removal with capturing CO_2_ from flue gas may provide an environmentally and economically useful system to reduce greenhouse gas emission. Numerous studies have demonstrated that wastewater grown algae has higher photosynthetic efficiencies and productivities when CO_2_ is added to the culture (FitzGerald and Rohlich, 1964; Heubeck et al., 2007; Kuo et al., 2016; Chaudhary et al., 2018). Bush et al. (1961) concluded that maximum algal productivity couldn't be gained unless CO_2_ was bubbled into algae wastewater pond. An increase in pH due to photosynthetic activity in any cultivation system can inhibit the optimum growth of microalgae. Addition of CO_2_ to wastewater treatment HRAP systems makes a balance in the acidity of media and thus neutralizes the effect of increasing pH. By sparging the system with CO_2_, the system can maintain an optimal pH in the range of 7.5–8.

### Energy Efficiency of Microalgae-Based Wastewater Treatment

Wastewater can potentially be converted into fertilizer. Unfortunately, a significant amount of these potential fertilizers is lost as untreated or partially treated wastewater worldwide. Synthetic agricultural fertilizers such as urea, di-ammonium phosphate, and potash are estimated to worth $420, $480, and $400 per ton, respectively (Komolafe et al., 2014). An added benefit of algae-based fertilizer is its ability to retain moisture in the soil and there is a slow release of N and P over time as needed by the terrestrial plants and trees. Thus, the ability of microalgae growing in wastewater to convert pollutants to a valuable biomass is of economic importance. In contrast, traditional wastewater treatment methods are dependent on the high cost of energy and treatment chemicals.

For example, aerobic decomposition of dissolved and particulate organic matter by bacteria in the sewage needs a high amount of oxygen. The energy input in the mechanical approaches is also high (45–75% of total energy costs). In addition, the process of nitrification added to an activated sludge wastewater treatment plant would increase the amount of energy by 60–80% (Maurer et al., 2003). The nutrient uptake rates for nitrogen in microalgae-based wastewater treatment systems can reach 24 kg N/ha/day and 3 kg P/ha/day. This is based on an assumed productivity of 30 g/m^2^/day algal biomass (dry weight). Overall the CC and O&M costs for an algae-based wastewater treatment system are far lower than comparable costs using conventional mechanical and chemical treatment technologies.

In addition to the afore-mentioned economical merits, treating wastewater with microalgae is essential for capturing CO_2_. A microalgae-based CO_2_ bio fixation requires a cost efficient system (i.e., HRAPs) and a low-cost nutrients source (i.e., wastewater). This suggests that CO_2_ removal could occur in both photobioreactors and HRAPs, making a synergistic integration (de Godos et al., 2010). A HRAP system using sunlight, bacteria and photosynthesis compared to the electromechanical treatment in a conventional oxidation pond reduces 100 to 200 tons of CO_2_ per ML of treated wastewater (Green et al., 1995; Benemann and Pedroni, 2003). An additional 100 to 200 tons of CO_2_ per ML could be abated due to the algal assimilation of nitrogen (Shilton, 2005).

### The Role of Integration in Reducing Fertilizer-Caused CO_2_ Emission

Typically, the production of one ton of nitrogen and phosphate fertilizers will release 3.15 and 1.39 tone CO_2EQV_, respectively (West and Marland, 2002; Wood and Cowie, 2004). One kg of algae containing 7%N and 0.8%P used as a fertilizer has the potential to reduce the CO_2_ emissions from conventional inorganic fertilizers by 0.23 kg CO_2EQV_ kg^−1^ Algae (West and Marland, 2002; Wood and Cowie, 2004).

### Use of the Integration Systems in Biofuel Generation

HRAP-based wastewater treatment is currently the most economical and environmental approach to produce algal biomass for conversion to biofuels. In this regard, technologies have been developed to raise the efficiency of microalgal CO_2_ fixation integrated with wastewater treatment, leading to value added products such biofuels (Kumar et al., 2010). By producing one ton algal biomass in HRAP-based wastewater treatment approximately 1.8 tons of CO_2_ can be fixed through photosynthesis (Benemann and Pedroni, 2003). Once converted to biofuel, this offsets emitted CO_2_ from the combustion of fossil fuel. For example, generating electricity from biogas methane accounts for mitigating CO_2_ emission to the atmosphere at 0.4 kg CO_2EQV_ kWh^−1^ and 1.0 kg CO_2EQV_ kWh^−1^ in comparison with natural gas and coal electricity generation. The use of algae-based biodiesel and bio-crude oil in place of petroleum-based diesel and heavy bunker fuel will result in a change in Green House Gas emission abatement of 2.68 kg CO_2EQV_ L^−1^ and 2.99 kg CO_2EQV_ L^−1^, respectively (New Zealand Ministry of Economic Development, 2007).

## Inherent Challenges Associated With Scale Up

Heavy metal ions such as Cd, Cr, Zn, and other ions, as well as organic chemical toxins such as surfactants, hydrocarbons, and biocides are all present in industrial wastewaters (Ahluwalia and Goyal, 2007). Effluents from metal processing industries as well as textile, leather, tannery, electroplating, and other industries have varying amounts of toxic metal ions. Because of low concentrations of N and P containing compounds as well as high levels of toxins, algal growth rates are lower in different industrial wastewaters as compared to those of domestic wastewater. Therefore, there is less potential for the large scale treatments of industrial wastewaters containing high levels of heavy metal ions for algal culture concurrent with CO_2_ removal. When the industrial wastewaters contain moderate levels of heavy metal ions, as well as P and N containing compounds at low concentrations they would be able to support algal growth. As previously mentioned, certain microalgal species are able to remove various types of metal ions. The main challenges that remain to be addressed by the researchers include but not limited to: industrial-scale capturing of carbon dioxide using microalgal species, the tolerances of specific algal strains grown in a wide variety and concentration of heavy metal ions in industrial wastewaters, and the numerous parameters to be optimized for simultaneous removal of CO_2_ and treatment of wastewaters containing heavy metal ions.

## Conclusion

Microalgae cultivation is one of the most efficient approaches to remove CO_2_ and each cultivation system has its advantage and drawbacks. Thus, selection of the best strategy strongly depends on the purpose, scale, microalgae species, and cost. However, open ponds appear to be more economically feasible than photobioreactors. In addition to CO_2_-biofixation, microalgae have been shown to be highly efficient in wastewater treatment. HRAP can provide efficient tertiary-level wastewater treatment compared to electromechanical wastewater treatment technologies and at a far lower cost. While it is possible to separately use microalgae for any of the processes of CO_2_ biofixation, wastewater treatment, or biofuel production, it would be more efficient to integrate all of these processes. Integration of microalgae-based CO_2_ fixation, biofuel production, and wastewater treatment could be considered multi-faceted approaches to manage important environmental challenges. It is a green and sustainable tool for reducing the production costs of bio-diesel, agricultural fertilizers, wastewater treatment, and flue gas treatment. Finally, it may lead to a reduction in greenhouse gases in an efficient way. In the future, large-scale algae-based treatment programs could minimize the current technical/economic barriers of microalgae-based bio-fuel production.

## Data Availability

All datasets generated for this study are included in the manuscript and/or the supplementary files.

## Author Contributions

This work is part of the M.Sc. thesis that MM did under the supervision of HA. HP and SL were involved in advising MM during her M.Sc. period. PR also read the manuscript several times and provided very detailed comments to improve the quality of the selected materials.

### Conflict of Interest Statement

The authors declare that the research was conducted in the absence of any commercial or financial relationships that could be construed as a potential conflict of interest.

## Footnotes

^1^http://www.esrl.noaa.gov/gmd/ccgg/trends/global.html
